# Crossing Latitudes—Long-Distance Tracking of an Apex Predator

**DOI:** 10.1371/journal.pone.0116916

**Published:** 2015-02-11

**Authors:** Luciana C. Ferreira, Michele Thums, Jessica J. Meeuwig, Gabriel M. S. Vianna, John Stevens, Rory McAuley, Mark G. Meekan

**Affiliations:** 1 The UWA Oceans Institute, School of Animal Biology, The University of Western Australia, Crawley, Western Australia, Australia; 2 Australian Institute of Marine Science, Perth, Western Australia, Australia; 3 Centre for Marine Futures, The University of Western Australia, Crawley, Western Australia, Australia; 4 CSIRO Marine and Atmospheric Research, Hobart, Tasmania, Australia; 5 Department of Fisheries, Government of Western Australia, WA Fisheries and Marine Research Laboratories, Perth, Western Australia, Australia; Institut Maurice-Lamontagne, CANADA

## Abstract

Tiger sharks (*Galeocerdo cuvier*) are apex predators occurring in most tropical and warm temperate marine ecosystems, but we know relatively little of their patterns of residency and movement over large spatial and temporal scales. We deployed satellite tags on eleven tiger sharks off the north-western coast of Western Australia and used the Brownian Bridge kernel method to calculate home ranges and analyse movement behaviour. One individual recorded one of the largest geographical ranges of movement ever reported for the species, travelling over 4000 km during 517 days of monitoring. Tags on the remainder of the sharks reported for shorter periods (7-191 days). Most of these sharks had restricted movements and long-term (30-188 days) residency in coastal waters in the vicinity of the area where they were tagged. Core home range areas of sharks varied greatly from 1166.9 to 634,944 km^2^. Tiger sharks spent most of their time in water temperatures between 23°-26°C but experienced temperatures ranging from 6°C to 33°C. One shark displayed seasonal movements among three distinct home range cores spread along most of the coast of Western Australia and generalized linear models showed that this individual had different patterns of temperature and depth occupancy in each region of the coast, with the highest probability of residency occurring in the shallowest areas of the coast with water temperatures above 23°C. These results suggest that tiger sharks can migrate over very large distances and across latitudes ranging from tropical to the cool temperate waters. Such extensive long-term movements may be a key element influencing the connectivity of populations within and among ocean basins.

## Introduction

Throughout human history, top-order predators have been disproportionately and negatively affected by anthropogenic activities, both directly through human behaviour such as hunting and indirectly by alteration of habitat and depletion of their food resources [1–4]. We now know that the ecological impacts of our elimination of top-order predators can be severe, leading to shifts in the composition and resilience of ecosystems through processes such as mesopredator release and trophic cascades [3,5,6]. Globally, the removal of sharks due to fishing has accelerated rapidly in recent decades, with the result that many species are now threatened with or vulnerable to extinction in many regions [7–12]. There is a growing recognition of the importance of sharks as keystone species in the structuring of marine ecosystems through their influence on species composition, biomass and the trophic roles of prey assemblages [10,13–15]. However, we remain largely unaware of some of the most basic aspects of the ecology of many species, including movement patterns and habitat requirements. As top-order predators, sharks tend to have large body sizes [16] and thus generally require large areas in which to forage. For this reason, they are likely to undergo long distance (10–1000s km) movements that could bring them into contact with multiple habitats, ecosystems and anthropogenic threats [16–18].

Tiger sharks (*Galeocerdo cuvier*) are one of the largest sharks, growing to over 5 m [19–21] and the species is both an apex predator and scavenger that occurs in most tropical and warm-temperate marine ecosystems. They feed on a wide array of prey [20,22–29], on which they exert both lethal and behavioural risk effects [30]. Globally, anthropogenic threats to populations of tiger sharks include commercial fisheries [7,31–34] and illegal, unreported and unregulated (IUU) fishing [9]. Moreover, they have been targeted by shark control programs as a species potentially dangerous to humans [35–40] and evidence of declines in populations of tiger sharks have been reported by beach meshing programs in some areas of Australia [21,41]. However, while the species is classified as “Near Threatened” by the International Union for the Conservation of Nature (IUCN) due to evidence of declines in some populations [42], broad-scale trends in abundances are still unknown.

Given the anthropogenic threats to these apex predators, information on their movement behaviour, particularly over large spatial and temporal scales (100s—1000s of km, months to years) is essential for the development of appropriate management and conservation strategies [43–46]. To date, most information on the horizontal movements of tiger sharks is available from SPOT and PAT satellite tracking studies conducted at relatively small temporal scales (<1 year; e.g. [47–51]). The information provided by SPOT transmitters is usually limited by the relatively short intervals that sharks spend on the surface, which results in low numbers of location estimates, obtained at irregular intervals and typically with low spatial resolution [47,49]. Additionally, physical damage, biofouling and premature shedding of tracking devices are also widely reported [47,48,52,53], resulting in short and sparse location data sets. For PAT tags in particular, deployment periods are commonly much shorter than programmed due to tag damage and biofouling that may cause premature release of the tag. Longer-term tracking studies have used passive acoustic telemetry to monitor movements of tiger sharks (e.g. [51,54–56]), but these are limited by the detection range of receivers and the number and scale of receiver arrays [57]. Overall, these studies have shown high individual variability in movement patterns of tiger sharks, with some degree of residency in particular habitats interspersed with occasional forays into the open ocean [47,48,51].

Due to the short duration of most tracking datasets it is unclear whether the wider movements across scales of 1000s of km, typical of other large sharks such as basking (*Cetorhinus maximus*), white (*Carcharodon carcharias)* and whale sharks (*Rhincodon typus)* [58–64] might also exist for tiger sharks. Our study reports on the results of the deployment of eleven satellite transmitters on tiger sharks, one of which produced one of the longest duration tracks ever recorded for the species (517 days). In combination with the results from the other deployments, we examined horizontal and vertical movements of tiger sharks and quantified habitats of high and low use. We hypothesise that tiger sharks display a mix of both restricted and transient movement, and that residency patterns will be driven by water temperature and bathymetry.

## Material and Methods

### Ethics statement

This project was conducted under permit number SF6104, WA Fisheries permit 2007–30–32, and ethics approvals A07035 (Charles Darwin University Ethics Committee) and DPIW 7/2007–08.

Tiger sharks were caught using longlines off Ningaloo Reef, Western Australia, in June 2007, August 2008 and May-June 2010. Between 118 and 350 hooks were set at approximately 10 m intervals along one to five demersal longlines deployed on the seaward side of the reef each day. Longlines were usually deployed at dawn, with a few deployments at dusk. Short soak times of between 2.2 h to 5.2 h were used to maximise the survival rates of captured sharks. One shark (Shark 7) was caught by rod and reel between longline sets. Hooked sharks were brought on deck or restrained in a stretcher at the stern of the vessel, measured and sexed. Eleven tiger sharks were instrumented with fin-mounted satellite-linked transmitters (SPOT4, SPOT5 or SPLASH tags, Wildlife Computers, Redmond, Washington, USA), however, three did not report any data after they were deployed (Table 1). All reporting transmitters relayed satellite positions via the ARGOS satellite network and time-at-temperature histograms in 14 user-defined temperature ranges from 0°C to 60° (± 0.2°C). Two SPLASH tags (Table 1) also relayed summaries of time-at-depth with user-defined depth ranges of 0–600 m and 0–800 m (± 0.5 m).

**Table 1:** Details of satellite transmitter deployments on tiger sharks.

ID	Fork length (cm)	Sex	Date deployed	Location	Type	Duration of transmissions (days)	Locations day^-1^ (mean ± sd)
1	145	F	19/06/2007	23.32°S 113.71°E	SPOT	7	0.75 (±1.16)
2	276	F	21/06/2007	22.97°S 113.76°E	SPLASH	14	0.92 (±1.50)
3	179	F	17/08/2008	21.86°S 113.96°E	SPOT	105	0.18 (±0.43)
4	214	F	19/08/2008	22.38°S 113.73°E	SPOT	70	0.54 (±0.83)
5	222	F	19/08/2008	22.42°S 113.71°E	SPOT	517	0.43 (±0.95)
6	333	M	27/05/2010	23.07°S 113.70°E	SPOT	191	1.86 (±1.66)
7	224	M	2/06/2010	21.58°S 114.52°E	SPOT	38	0.60 (±0.72)
8	240	F	30/05/2010	22.27°S 113.72°E	SPLASH	154	0.04 (±0.19)
9	155	F	19/06/2007	23.35°S 113.72°E	SPOT	Did not report	
10	252	F	21/06/2007	22.97°S 113.76°E	SPLASH	Did not report	
11	254	F	20/08/2008	22.66°S 113.60°E	SPLASH	Did not report	

Position estimates were provided by ARGOS with an associated error (Location Class (LC) of 3 (<250 m), 2 (250–500 m), 1 (500–1500 m), 0 (>1500 m), A and B (not specified), www.argos-system.org). A small amount (0.5%) of location points were excluded or substituted by the secondary location estimate reported by ARGOS because they were obviously erroneous, i.e. they were well beyond the bounds of possible distances the shark could have travelled based on both earlier and later location estimates for the track. An analysis of the travel speed found that these erroneous locations required a travel speed of >1000 km per day. More advanced filtering methods were attempted, such as a Bayesian state-space model, however, the models did not converge, probably due to the sparseness of the data.

We calculated the home range of tiger sharks using the Brownian Bridge kernel method using the *adehabitatHR* package in R software V2.15.3 [65,66]. This method takes into account not only the shark locations, but also the path travelled by the animal between successive locations [67,68] by applying a conditional random walk to model the expected path between locations. Two smoothing parameters were set: sig1, which controlled the width of the “bridge" connecting successive positions (this is the Brownian Bridge motion variance parameter—BMV); and sig2, which was related to the imprecision of the positions. Values of sig1 were chosen using the function *liker* that implemented the maximum likelihood approach developed by Horne [68] and sig2 was set at 1.9 km, the median ARGOS location error across pooled position classes [69]. Tracks from Sharks 1 and 2 were removed from the analysis due to their very short length (7 and 14 days respectively). Because of the possibility of effects of tagging on behaviour, we assumed that the first two weeks might not be representative of the shark’s natural behaviour [70]. Location estimates from Sharks 3 and 8 were particularly sparse (0.18 and 0.04 locations per day respectively), and Shark 3 had a very high Brownian Bridge motion variance parameter compared to the scale of the shark’s movements (sig1 = 822.5). Consequently, these tracks were not included in further analyses of home range.

The proportion of observations in each temperature and depth bin was calculated from time-at-temperature and time-at-depth histograms provided by the tag to determine thermal and depth range of all sharks. For one shark (Shark 5), time-at-temperature histograms associated with latitude/longitude information were used to assess variation in the proportion of time at temperature. Water temperature profiles were constructed from data downloaded from IMOS floats (IMOS, http://imos.aodn.org.au/imos/) located in the vicinity of position uplinks within a week of the time the uplinks were recorded (South coast of WA: 35.378°S, 119.531°E; North coast of WA: 17.604°S, 117.657°E). Maximum possible diving depth of Shark 5 was estimated by comparing the minimum temperature registered by the shark’s tag with profiles of water temperature from IMOS floats in the same region where the shark was resident. The maximum depth of descent was assumed to be the greatest depth in the water temperature profile where the minimum temperatures reported by the tag and those of the water temperature profile were the same. For the track with multiple home range cores (Shark 5), movement patterns were categorised as within and outside the 25% utilisation distribution. We then used generalised linear models with a binomial distribution and a logit link function to assess the relationship between the probability of the shark being in a home range core and water temperature, bathymetry and region of the WA coast (north—latitude < 24°S and south—latitude >24°S). We were not able to fit all three explanatory variables in one model as there was no overlap of the temperature ranges between the two regions. Consequently, we fitted a model to examine the probability of being in a home range core in relation to bathymetry and region and two separate models (using the data from north and south coasts, respectively) to examine the probability of the shark being in a home range core in relation to sea surface temperature. To address the autocorrelation present in the data we used a matched-block bootstrap sampling for all models with replacement procedure [71,72] that resampled blocks of data randomly and then recombined them in a random order, creating a bootstrapped dataset that minimized the effect of autocorrelation [71–73]. Model fitting was applied to 100 bootstrapped samples and model selection used the sample-corrected Akaike’s information criterion (AIC_c_), AIC_c_ weight (_*w*_AIC_c_), and percent deviance explained (%DE) [74,75]. Bathymetry data with a grid resolution of 2’ from ETOPO1 database hosted by the NOAA was obtained by the R software package *marmap* [76]. Daily Sea Surface Temperature was obtained through the daily Optimum Interpolation Sea Surface Temperature (OISST) analysis [77] on a 0.25 degree latitude/longitude grid from NOAA’s National Climatic Data Centre (ftp://eclipse.ncdc.noaa.gov/pub/OI-daily-v2/NetCDF).

## Results

The time that data was received from the tagged sharks varied greatly among individuals (7 to 517 days; Table 1). 49% of locations were from ARGOS Location Class B, 20% were from Location Class A and only 31% of locations were within Location Classes 3–0. Four satellite transmitters provided data for less than 100 days but even the longest deployments had long periods of no transmission. For example, Shark 5 had a period of 118 days between May and September 2009 when no location data were transmitted, while Shark 8 did not transmit data between July and October 2010.

**Fig 1:**
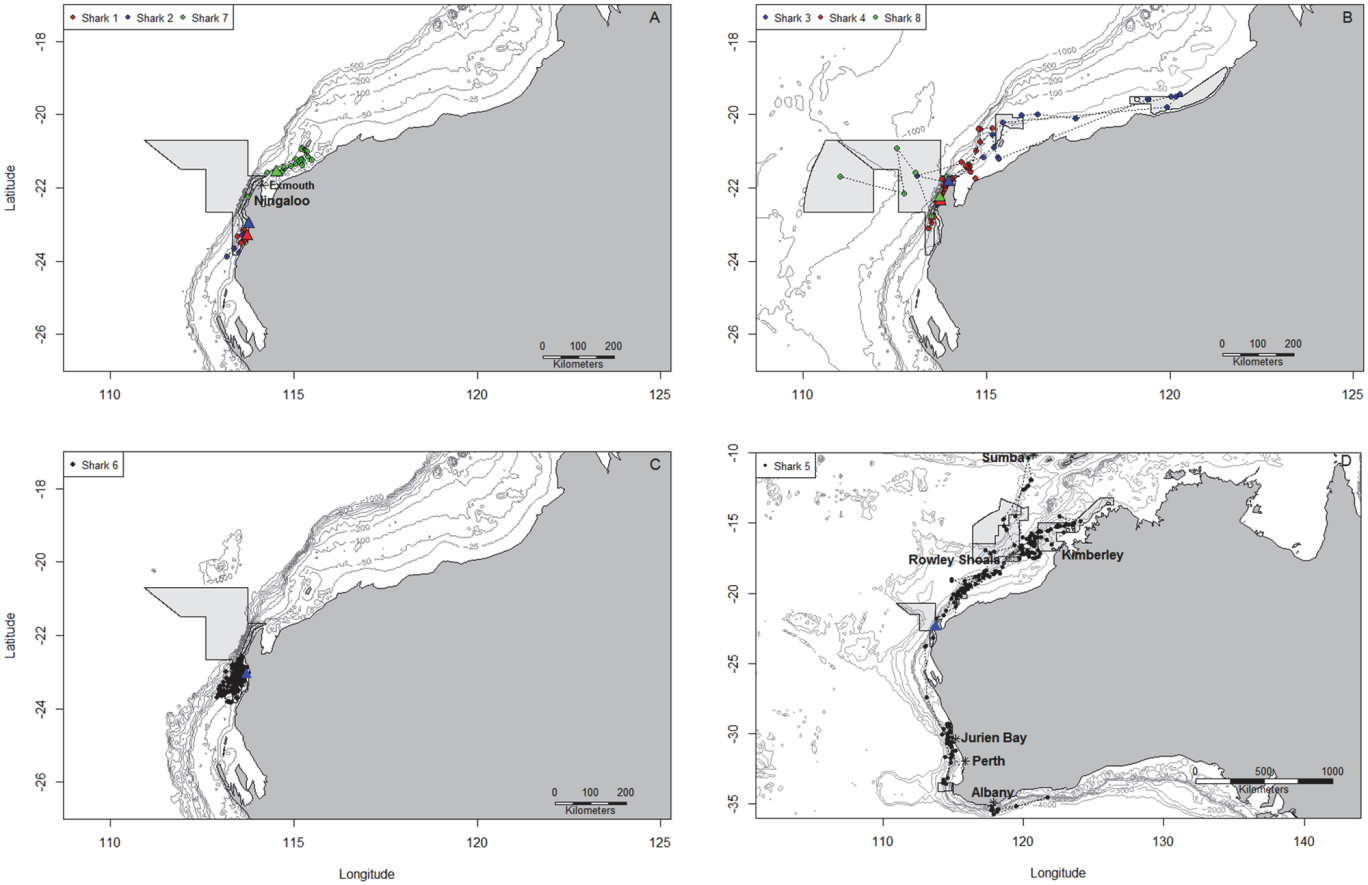
Movement patterns of tiger sharks in Western Australia. Maps show location uplinks of 8 tiger sharks. Triangles indicate tagging location and grey polygons indicate Commonwealth Marine Reserves.

Transmitters deployed on three sharks (1, 2 and 7) provided data for only 7, 14 and 38 days respectively. During this time they occupied waters with average depths of 42.2 m (± 26.8 m sd) and their movements were restricted to the vicinity of where they were initially tagged (Fig. 1A) on the shelf. Three sharks (3, 4 and 6; Fig. 1B–C) stayed within shelf waters that averaged 117.5 m deep (± 113.0 m sd). Shark 6’s movements were restricted to a relatively small area (1166.9 km^2^) off the Ningaloo Reef for six months (Fig. 1C). Shark 8 moved 303.4 km from the point of first capture and tagging into waters over 5000 m deep, where the last data transmission was received (Fig. 1B). There were six position estimates in this area and the data were very limited for the duration of tracking (154 days between tagging and the last uplink). One shark (Shark 5) ranged over 4000 km and apart from one period of approximately three and a half months, provided relatively frequent transmissions over 517 days of monitoring (Fig. 1D).

**Fig 2:**
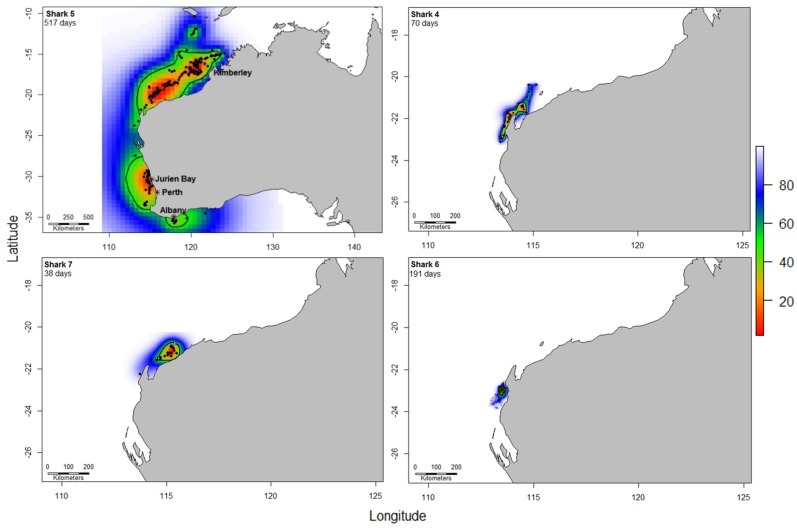
Home range of tiger sharks. The utilisation distribution calculated using the Brownian Bridge kernel method. Black line represents the 50% Brownian Bridge home range distribution.

The kernel utilisation distributions of all sharks indicated movement between coastal regions and islands or atolls off the north coast of Western Australia, with predominant use of coastal waters (Fig. 2). The 50% kernel utilisation distributions varied greatly in area from 1166.9 km^2^ to 634,944 km^2^ among sharks (Fig. 2). Overall, a total of 56.7% of all locations received from the satellite transmitters were within the Commonwealth Marine Reserve network. All sharks had some locations inside marine reserves and six of the sharks had 50% or more locations inside reserves (Fig. 1).

Shark 5, a 222 cm female, was monitored for 517 days and ranged from 10.4°S to 35.8°S of latitude and from 113.0°E to 124.1°E of longitude (Fig. 1D). The transmitter was deployed on this shark at Ningaloo Reef and the shark then moved along the 500 m bathymetric contour to the Rowley Shoals and Kimberley region. It then made a path to Sumba Island, Indonesia, and returned, crossing ocean depths of 5 km and covering a distance of more than 1000 km in 2 weeks. In December 2008 the shark moved south, traveling to waters off Jurien Bay and Perth between January-February 2009. Between April and May 2009 the shark rounded Cape Leeuwin with transmissions clustering off Albany. After a period of no transmissions (118 days), the tag then started to transmit again in September when the shark moved towards the north, returning again to Perth/Jurien Bay in January 2010. The shark had three distinct areas of 50% Brownian Bridge kernel utilisation that included a large area off the north coast of Western Australia, another off Jurien Bay and Perth and a smaller one off the coast of Albany (Fig. 2).

**Fig 3:**
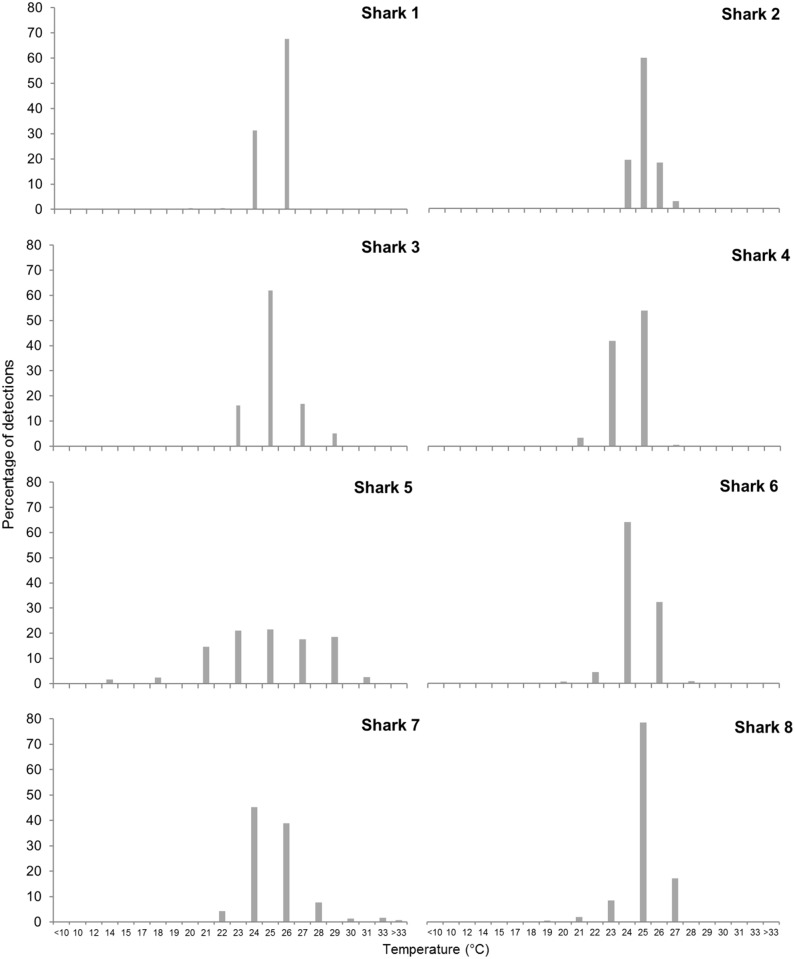
Time spent in each temperature bin. Plots show the percentage of time spent within the specified temperature bins for each tiger shark (ID number in the top right hand corner of each plot).

**Fig 4:**
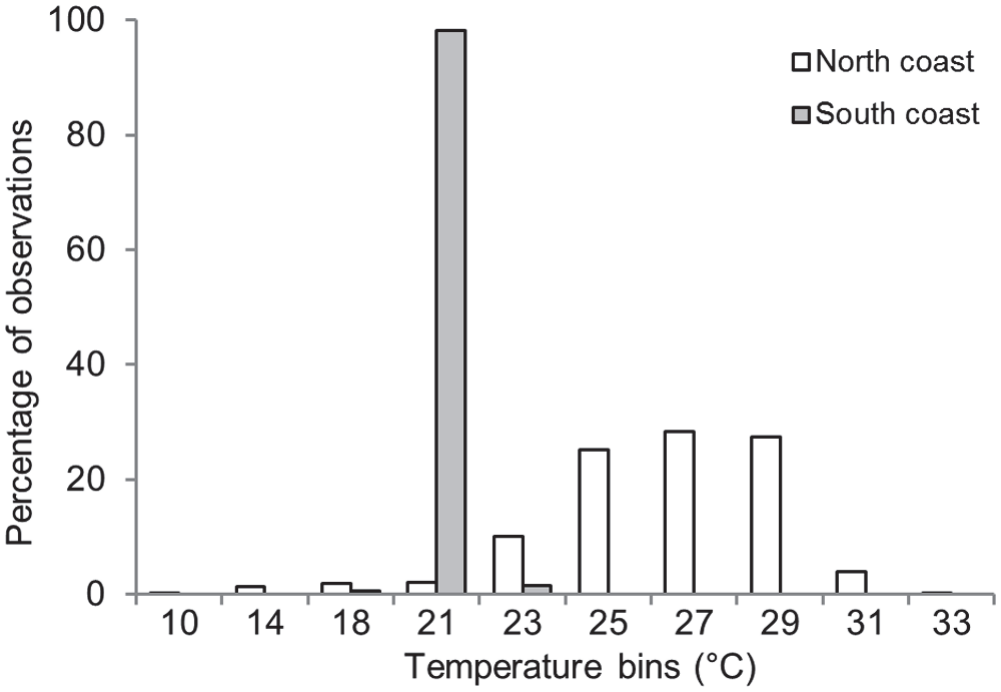
Latitudinal variation of temperature profiles for Shark 5. Percentage of time spent in each temperature bin at each region of Western Australia for Shark 5.

Temperatures experienced by sharks ranged from 6°C to 33°C. Overall, tiger sharks spent most of their time in temperatures between 23°-26°C (Fig. 3). The shark with the widest latitudinal range of movements (Shark 5) also experienced the greatest range of temperatures (from 10°-33°C), but spent approximately 78% of time in temperatures between 23°-29°C. It also experienced different ranges of temperature in each home range (Fig. 4). Off the Kimberley and Pilbara coasts (10°-24°S of latitude) the temperature range experienced by the shark was much greater (10°-33°C) than when the shark visited the south coast off Albany (35°S of latitude; 18°-23°C).

**Fig 5:**
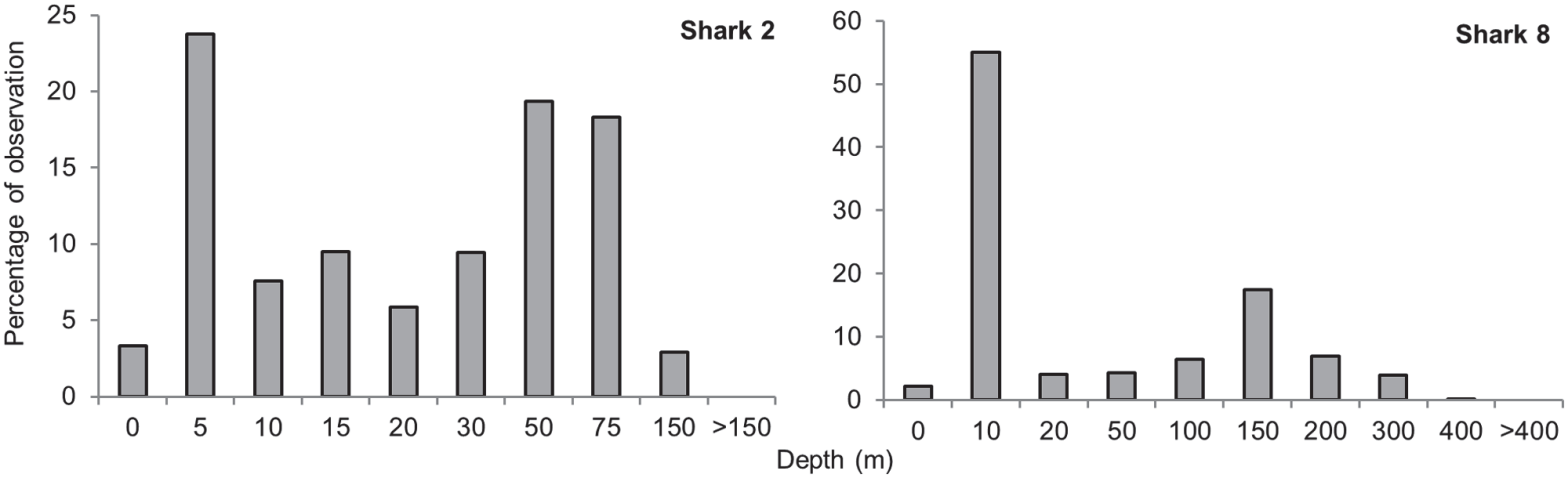
Time spent in each depth bin. Percentage of time spent in each depth bin for two tiger sharks tagged with SPLASH tags.

Time-at-depth histograms of the two sharks with SPLASH tags showed that these individuals differed greatly in depth range (Fig. 5). The first (Shark 2) did not dive deeper than 150 m, with 27% of observations between the surface and 5 m and 97% in water depths up to 75 m. Locations for this shark were all close to the coast in areas around the 100 m isobath. The second (Shark 8), which moved to pelagic waters experienced a greater range of depths. Though this shark spent 57% of time between the surface and 10 m, it had a maximum recorded depth of 400 m, with approximately 20% of time spent at 150 m.

**Fig 6:**
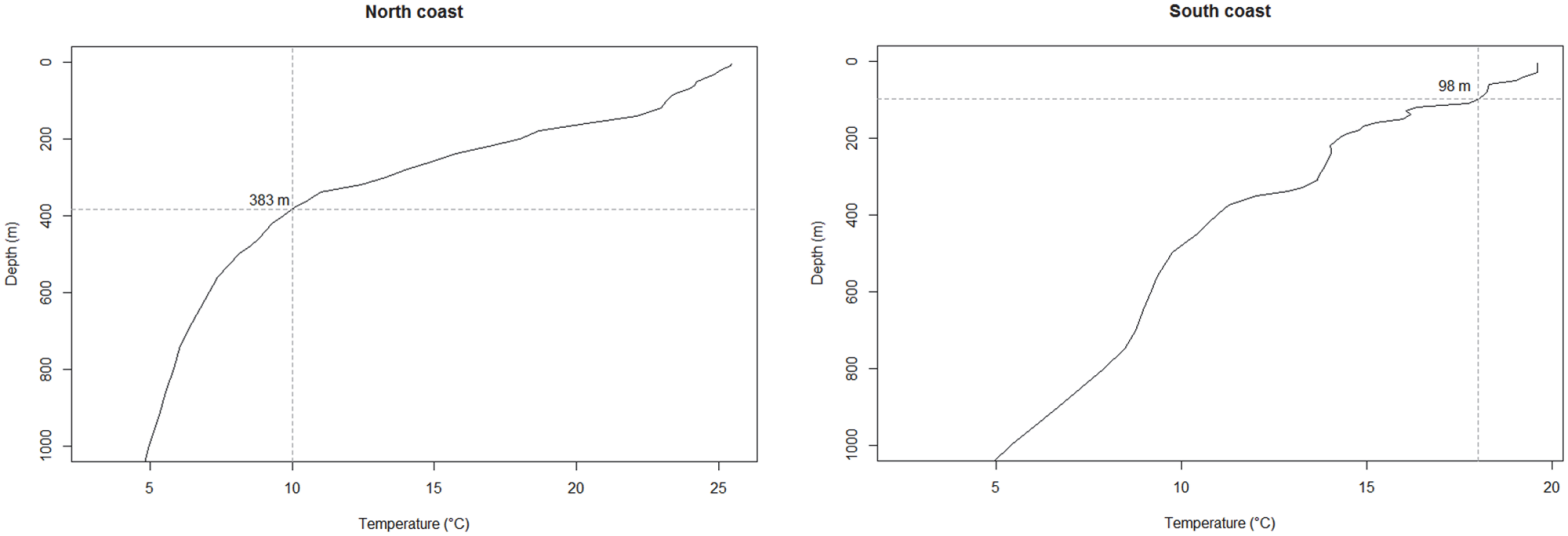
Vertical profiles of water temperature. Plots show temperature profiles recorded by Argo floats of the north (17.6°S 117.6°E) and south (35.78°S 119.5°E) sections of the WA coast. Vertical lines represent the minimum temperature reported by the shark’s satellite transmitter and horizontal lines represent the estimated diving depth.

Water temperatures reported by Shark 5, which was tracked for 517 days, were used to estimate diving depths. Analyses of the vertical profiles of water temperature in each region where location data were recorded indicated that the shark was diving up to 380 m in tropical latitudes, but not descending below 100 m in temperate latitudes (Fig. 6). Sea surface temperatures were consistent with a strong Leeuwin Current that supplied a warm mass of water around the Cape Leeuwin and Albany coasts with temperatures of 19°-23°C during the time locations were registered in this region (S1 Fig.). Here, the shark spent 82% of time in waters between 21–23°C (Fig. 4), indicating that it remained relatively close to the surface.

**Fig 7:**
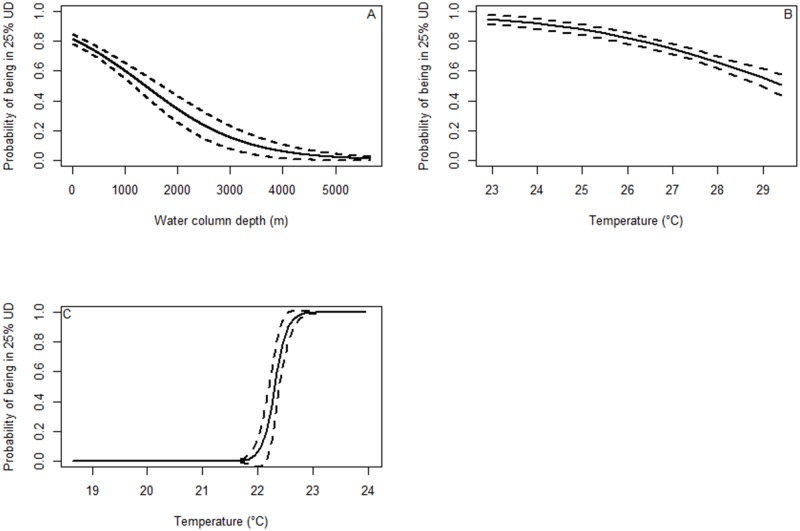
Generalised linear model for Shark 5. Generalised linear model (GLM) predicted probabilities (solid line) of a shark being in a 25% utilisation distribution in relation to (a) water depth, (b) water temperature in the north and (c) in the south. Dotted lines show the standard error.

The highest ranking model describing whether the shark remained in a 25% home range core (i.e. remained resident and did not switch to transit behaviour), included water depth only, and explained 34.5% of the deviance in the data set (Table 2). There was a negative relationship between the probability of the shark being in a 25% home range core and water depth, meaning the shark was more likely to be resident (in a home range core) in the shallower areas and less likely to remain resident in deeper waters (Fig. 7). Even though the tiger shark crossed areas over 5000 m deep while migrating, it had low probability of being in a home range core in deeper water (> 1000 m).

**Table 2:** Ranked Generalised Linear Models with bootstrap sampling of the probability of a shark being in a 25% utilisation distribution explained by bathymetry (Depth) and region of the coast (Region).

Model	LL	df	AIC_*c*_	_*w*_AIC_*c*_	%DE	LL.25	LL.75	_w_AIC_c_.25	_w_AIC_c_.75	AIC_c_.25	AIC_c_.75	%DE.25	%DE.75
Home _˜_ Depth	-82.456	2	168.967	0.508	34.49	-124.637	-101.77	0.001	0.317	207.595	253.328	5.75E+00	20.347
Home _˜_ Depth + Region	-82.010	3	170.131	0.284	34.84	-122.153	-97.006	0.167	0.5143	200.125	250.417	6.85E+00	23.756
Home _˜_ Depth*Region	-81.283	4	170.751	0.208	35.42	-117.994	-95.781	0.221	0.649	199.749	244.173	9.53E+00	25.453
Home _˜_ Region	-125.803	2	255.661	0.000	0.05	-134.792	-118.812	0	0.001	241.678	273.639	2.98E-01	4.687
Home _˜_ 1 (Null)	-125.863	1	253.744	0.000	0.00	-137.028	-124.8	0	0	251.619	276.075	-1.91E-14	0

Maximum log-likelihood (LL), degrees of freedom (df), Akaike’s information criterion corrected for small samples (AIC_*c*_), AIC_*c*_ weight (_*w*_AIC_*c*_), the % deviation explained (%DE), lower quantile LCI (25^th^ quantile) (25%) for LL.25, _*w*_AIC_*c*_.25, AIC_c_.25 and %DE.25 and upper quantile (75^th^ quantile) for LL.75, _*w*_AIC_*c*_.75, AIC_c_.75 and %DE.75.

Temperature explained 9.7% of the deviance in the probability of Shark 5 being in a home range core on the north coast and 44.5% in the south (Table 3). In both regions, the shark had a greater than 80% chance of being in a home range core in temperatures of 23–24°C, suggesting that this was a preferred temperature in both regions (Fig. 7).

**Table 3:** Ranked Generalised Linear Models with bootstrap sampling of the probability of a shark being in a 25% utilisation distribution explained by sea surface temperature (Temperature) at the north and south coast separately.

Model	LL	df	AIC_*c*_	_*w*_AIC_*c*_	%DE	LL.25	LL.75	_w_AIC_c_.25	_w_AIC_c_.75	AIC_c_.25	AIC_c_.75	%DE.25	%DE.75
North													
Home _˜_ Temperature	-94.845	2	193.756	0.999	9.67	-101.645	-80.5813	0.748725	1	165.2297	207.3572	0	0
Home _˜_ 1 (Null)	-111.514	1	212.024	0.000	0.00	-111.514	-91.553	0	0.251	185.128	225.05	2.184	25.802
South													
Home _˜_ Temperature	-16.535	2	37.337	1	44.54	-13.75475	0	1	1	4.267	31.7765	0	0
Home _˜_ 1 (Null)	-29.812	1	61.711	0.000	0.00	-32.895	-29.812	0	0	61.711	67.877	30.254	57.026

Maximum log-likelihood (LL), degrees of freedom (df), Akaike’s information criterion corrected for small samples (AIC_*c*_), AIC_*c*_ weight (_*w*_AIC_*c*_), and the % deviation explained (%DE), lower quantile LCI (25^th^ quantile) (25%) for LL.25, _*w*_AIC_*c*_.25, AIC_c_.25 and %DE.25 and upper quantile (75^th^ quantile) for LL.75, _*w*_AIC_*c*_.75, AIC_c_.75 and %DE.75.

## Discussion

Tiger sharks are typically considered to be residents of tropical and warm temperate habitats [20], but individuals appear on a seasonal basis in temperate or cool temperate waters [78]. The most extreme examples of this pattern are the occasional records of tiger shark catches in the waters of countries such as Iceland [79] and far off the south coast of New South Wales in Australia [80]. Such occurrences seem to be related to the influx of warm waters brought by western boundary currents such as the Gulf Stream and the East Australian Current [81–83]. Similarly, in Western Australia the Leeuwin Current flows southward bringing warm, low salinity water along the shelf into the cool temperate environments off Albany (35°S) [84]. In our study, one shark moved into temperate waters twice, both times during the summer months of January-February. This implies that such behaviour is most likely to be the result of directed and seasonal migrations, rather than simply a haphazard event. Other evidence to support this idea of regular southward movements of tiger sharks during the austral summer comes from data from beach protection programs, which show that tiger sharks are more common off the coast of New South Wales, a temperate region in the southeast of Australia, during summer [41,85], when the warm East Australian Current flows southward. A recent tagging study found that a tiger shark moved to warmer waters off Queensland during winter and went south to 37°S latitude during the austral summer [86]. Similarly, the abundance of tiger sharks in the Aliwal Shoals off South Africa increases during summer months [87].

The shark we tracked for 517 days had home range core areas in both the tropics (15°-20°S) and the cool-temperate coast off Albany (35°S). The movement of this shark to temperate waters off Perth occurred twice in consecutive years, both times during January. Overall, this track was the longest and covered one of the greatest range of latitudes recorded by satellite telemetry for the species. However, such movements are not entirely without precedent. For example, a fisheries tagging study recorded tiger sharks travelling more than 3,400 km in the Atlantic [88] and recent satellite tagging studies have shown tiger sharks swimming distances of over 1000 km in the South Pacific [51,86], and as far as 3500 km from the tagging site in the North Atlantic [89]. A study by Heithaus et al. [47] recorded a potential movement of a tiger shark of 8000 km from the point where it was tagged in Shark Bay, WA to the coast of South Africa. However, this result was inferred from a single low-quality position fix that could not confirm if the tag was still attached to the shark or determine if the position estimate was simply an artefact of the position estimation algorithm. In our study, associated water temperature data reported by the tag and multiple position fixes showed that the track of the shark was both reliable and that the tag remained deployed on the shark. In terms of geographic scale, the movements of this tiger shark were comparable to those of white sharks (*Carcharodon carcharias*), which in certain regions appear to display seasonal residency at cool temperate coastal locations, mostly in areas with high abundances of pinnipeds [90–93], interspersed with long oceanic migrations into the tropics [60–62,94].

The durations of residency and distances of migration of tiger sharks recorded by our study appear to be at least partly related to the length of tag deployment. When tags reported for a short time, movements of sharks tended to be restricted to one home range core within a relatively small area. However, longer-term tag deployments revealed a very different pattern, where periods of residency were interspersed with long distance (100–1000s km) movements that appeared both directed and predictable. However, some individuals appeared highly resident despite relatively long tagging records (up to 6 months) suggesting that two forms of movement may be present. In our study, home range cores (averaging 4,474.1 km^2^, excluding Shark 5) were consistent with earlier work that has shown movement patterns of tiger sharks off the east coast of Australia [86] and the Florida coast [89].

Similar to our results, most other studies of tiger sharks have also shown that the degree of residency varies greatly among individuals. Fitzpatrick et al. [55] found that some sharks remained in the area of Raine Island on the Great Barrier Reef throughout the year, while others ventured into the Coral Sea and to the Torres Strait and northern Great Barrier Reef. Werry et al [51] found that tiger sharks could reside year-round at the atolls of the Chesterfield Reefs, with other sharks venturing over 1000 km into the open ocean. Meyer et al. [48] found that some tiger sharks were resident on the French Frigate Shoals of Hawaii throughout the year, while others were recorded at this locality only when fledging seabirds were available as a food source during summer. It has been suggested that variations in movement behaviour of tiger sharks might be due to partial migrations, where only a part of the population would be transient and perform large scale movements while the other part would show resident behaviour [51,56]. Although our study was comprised of a small sample size, recent tagging studies in Florida, Bahamas, Australia and New Caledonia have consistently demonstrated that a few individuals can move over large distances while most of the remainder sharks show more restricted residency patterns [51,86,89]. Analyses involving larger datasets are needed to understand the characteristics of individuals that show these partial and/or complete migrations, to determine their prevalence and to identify the drivers that lead to differential patterns of movement.

Despite the number of satellite and acoustic telemetry studies now describing the horizontal movements of tiger sharks (approximately 10 to date) it is difficult to determine if there are consistent patterns in the life stage or sex of individuals that are likely to remain resident or migrate. Meyer et al [55] suggested that juveniles were more likely to display broad-scale patterns of movement than other components of the population, a pattern also recorded in Northeast Brazil [50]. The individual that we recorded moving the greatest distance on the WA coast was also a sub-adult (female). In contrast, Papastamatiou et al. [56] found that adult females were the most likely to migrate among the islands of Hawaiian Archipelago, a pattern they attributed to the movement of females to pupping sites. However, these researchers mostly relied on records generated from acoustic tracking and a receiver array in coastal waters, limiting the possibility of recording movements of sharks into the open ocean.

The tiger sharks we tagged showed preferences for temperatures between 23° and 26°C, consistent with surface waters off Ningaloo Reef [95], although the sharks experienced temperatures as low as 6°C. These lower temperatures suggest that sharks were descending below the thermocline (± 100–200 m depths at Ningaloo Reef; [96]). Data from a SPLASH tag deployed on Shark 8 recorded occasional transits to depths of 400 m, although time-at-depth histograms showed a preference by this shark for shallow waters (65% of time in depths < 50 m) within the mixed layer of the water column, similar to patterns reported by other studies [48–51,54,97,98]. For five sharks, location estimates at Ningaloo Reef with depths <15 m suggested that some sharks spent time in the shallow lagoon.

Shark 5 experienced a wide range of temperatures within a home range in the tropics, suggesting that it used the water column to depths of 380 m, well below the thermocline in this region [96]. When the shark moved to the south coast off Albany, it remained in waters above 100 m deep, implying that its vertical movements may have been constrained by water temperatures when in the cooler waters at more southern latitudes.

The long-term residency of tiger sharks in limited areas implies that they may have strong structuring effects on those ecosystems. A study conducted for 15 years [99] found that the presence of tiger sharks at Shark Bay had major influences on the behaviour, movement and feeding of prey such as dolphins, turtles and dugongs, but beyond this largely seagrass habitat, we have little idea of the role of these predators in environments such as coral or temperate reef systems. In addition to quantifying the residency patterns of tiger sharks, our study showed large-scale movements that have implications for conservation, since this behaviour may take them across management and national boundaries. In our study, more than half of the locations provided by satellite transmitters were within the Commonwealth Marine Reserves network, although our results also support the suggestion that such regional scale management zones are likely to only provide temporary protection for some parts of the population or life history stages [16,51,100,101]. The movement of sharks from Australia to Indonesian waters also shows that conservation of these animals will also depend on international cooperation to mitigate anthropogenic threats to the resilience of the species, such as the large IUU shark fishery in Australia’s northern waters and small-scale shark fishing industry in Indonesia to the north [9].

Future research efforts will be aided by technological developments of satellite transmitters and attachment methods. One such development, the Fastloc GPS, already exists, however is unavailable on fin-mounted devices. Fastloc tags are capable of acquiring the data required for a location fix in a much shorter period of time and with greater location accuracy than other types of satellite tags, improving both the frequency of location estimates and the accuracy of position fixes. Ultimately, there are two key goals that must be achieved in order to obtain a better understanding of the movement ecology of tiger sharks: firstly, improved attachment techniques for tags that allow both frequent uploads of position data and long deployments (> 1 year) and secondly, tagging of a larger number of animals and a wider range of life history stages of both sexes. Our study shows that these advances will be necessary to gain a better appreciation of the role of these animals in the ecology of marine ecosystems.

## Supporting Information

S1 Fig Sea Surface Temperature map for 16^th^ May 2009 with location uplinks for Shark 5. (TIF)
