# Supplementary figures and images for: Crossing Latitudes—Long-Distance Tracking of an Apex Predator
Source: PLoS One. 2015 Feb 11;10(2):e0116916. doi: 10.1371/journal.pone.0116916 (PMC4324986; doi:10.1371/journal.pone.0116916)

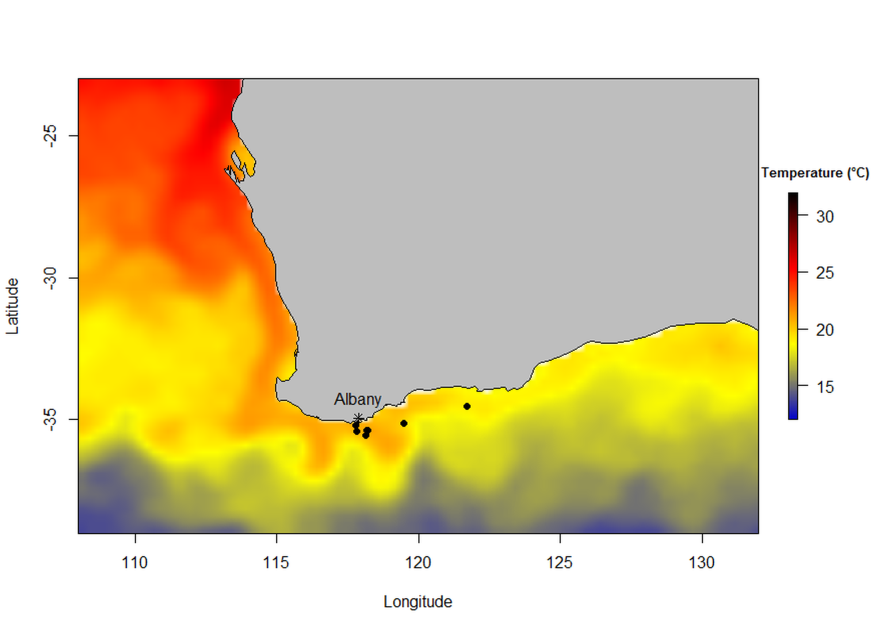

Supplement: S1 Fig — (TIF) [file pone.0116916.s001.tif]
